# Perspectives of key interest groups regarding supervised Consumption sites (SCS) and novel virtual harm reduction services / overdose response hotlines and applications: a qualitative Canadian study

**DOI:** 10.1186/s12954-024-01053-3

**Published:** 2024-07-27

**Authors:** Boogyung Seo, William Rioux, Adrian Teare, Nathan Rider, Stephanie Jones, Pamela Taplay, S. Monty Ghosh

**Affiliations:** 1 Department of Medicine, Faculty of Medicine & Dentistry, University of Alberta, 2J2.00 Walter C Mackenzie Health Sciences Centre, 8440 112 St. NW, Edmonton, AB T6G 2R7 Canada; 2 College of Medicine, University of Saskatchewan, Saskatoon, SK Canada; 3 Cumming School of Medicine, University of Calgary, Calgary, AB Canada; 4 Three Hive Consulting, Vancouver, BC Canada; 5 Grenfell Ministries, Hamilton, ON Canada; 6 Department of Internal Medicine, Faculty of Medicine & Dentistry, University of Alberta, Edmonton, AB Canada

**Keywords:** Supervised consumption sites, Overdose prevention sites, Virtual overdose response services, Spotting, Substance use, Overdose response hotlines and apps

## Abstract

**Background:**

Supervised consumption sites (SCS) and overdose prevention sites (OPS) have been implemented across Canada to mitigate harms associated with illicit substance use. Despite their successes, they still contend with challenges that limit their accessibility and uptake. Overdose response hotlines and apps are novel virtual technologies reminiscent of informal “spotting” methods that may address some of the limitations. Here, we strove to qualitatively examine the factors that may encourage or deter utilization of these virtual services and SCS.

**Methods:**

A total of 52 participants across Canada were recruited using convenience and snowball sampling methods. These included people with lived and living experience of substance use, family members of people with lived experience, healthcare providers, community harm reduction workers, and virtual harm reduction operators. Semi-structured telephone interviews were conducted and inductive thematic analysis was performed to identify the themes pertaining to SCS and virtual harm reduction.

**Results:**

Participants viewed overdose response hotline and apps as an opportunity to consume substances without being hindered by logistical barriers (e.g., wait times), fear of law enforcement, invasion of privacy, and more. They also noted that these virtual services provided more flexibility for clients who opt for routes of consumption that are not supported by SCS, such as smoking. Overall, SCS was perceived to be better than virtual services at facilitating social connection, providing additional resources/referrals, as well as prompt response to overdose.

**Conclusion:**

In sum, participants viewed SCS and virtual services as filling different needs and gaps. This study adds to a growing body of literature which informs how virtual harm reduction services can serve as useful adjunct to more standard harm reduction methods.

## Introduction

Community-led harm reduction and addiction supports have scaled up across Canada [1, 2] to address the overdose crisis that has seen more than 40 000 opioid toxicity deaths between 2016 and 2023 [3]. One of these efforts include supervised consumption sites (SCS), which are federally sanctioned facilities that offer safer spaces for people to consume pre-obtained drugs in the presence of trained staff [4]. Today, there are 39 sites operating across Canada with an estimated number of 2700 visits each day [4]. In recent years, provinces have also developed their own urgent public health sites or overdose prevention sites (OPS), which are designed to provide temporary support to local communities that require more adequate access to harm reduction supplies and services [5, 6]. However, SCS and OPS face numerous challenges to its implementation and operation [7, 8] despite the growing body of evidence demonstrating their effectiveness and ability to reduce harms associated with substance use [9, 10]. Furthermore, solitary substance use continues to pose challenges in curbing overdose mortality rates [11, 12], with recent data from Health Canada revealing that 78% of acute toxicity events still occur in private residences for those who are not experiencing homelessness [13].

**Fig. 1:**
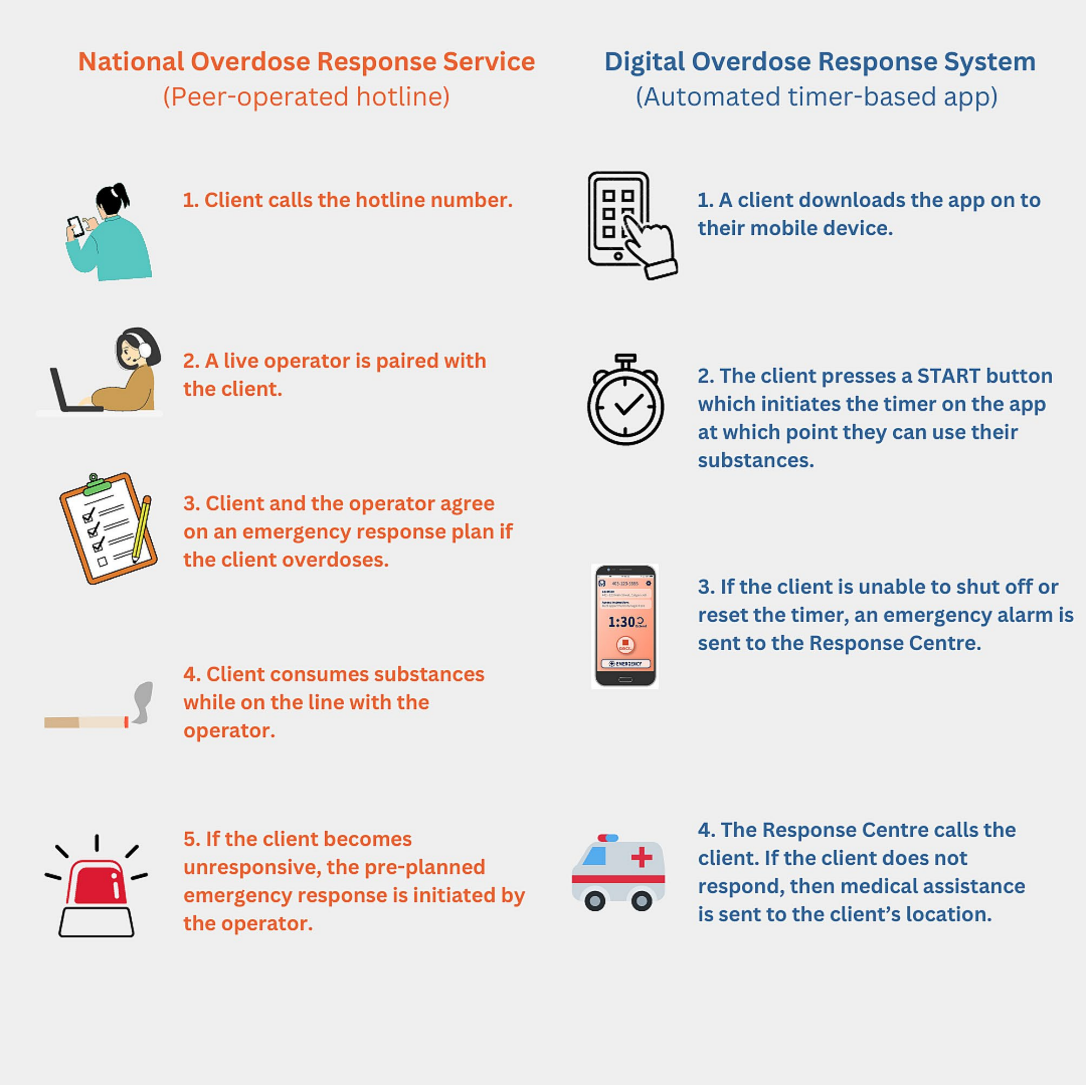
A step-by-step illustration of how a client may use peer-operated hotline and automated timer-based services

Spotting is an informal overdose spotting technique in which individuals can consume substances under the virtual supervision of a trusted individual (e.g., a friend, or family member) [14, 15]. It ensures autonomous and confidential substance use without being subjected to stigma or adherence to rigid organizational policies [14, 15]. Born out of these grassroots efforts, formalized virtual harm reduction services such as overdose response hotlines and apps have emerged to prevent fatal overdoses, especially for those who prefer to use substances alone and/or do not have immediate access to a SCS [16–19]. These services are alternatively known as Mobile Overdose Response Services (MORS) [20]. Certain services like the National Overdose Response Service (NORS) [21, 22] and BRAVE app [23] in Canada, as well as Never Use Alone in the United States, connect peers to an operator who can initiate a personally tailored and pre-planned emergency response (e.g., calling a friend/family member or emergency medical services) when an overdose is suspected [17]. In addition, British Columbia and Alberta have implemented their own timer-based mobile apps, called the LifeGuard [24] and the Digital Overdose Response Service (DORS) [25], respectively. These services activate an emergency response when the individual becomes unresponsive and is unable to shut off or renew the timer; however, they do not refer clients to a live operator. Figure 1 illustrates how some of these services function. To date, the authors are not aware of studies that have qualitatively compared these novel virtual services to more standard harm reduction strategies, such as SCS. This study aims to identify some of the strengths as well as the gaps in the current provision of virtual and in-person services to inform the development of these programs in the future.

## Methods

### Participants

The selected key interest groups who participated in the study consisted of: people with lived or living experience of substance use, family member of people with lived experience, healthcare providers, community harm reduction workers, and virtual harm reduction operators. Both convenience and snowball sampling were employed to recruit participants using existing networks known to the research team and one overdose response hotline service and an overdose response app services operations team. The study was open to any individuals residing in Canada who were 18 years of age or older, able to communicate effectively in English, and able to provide informed verbal consent.

### Semi-structured interviews

A semi-structured interview guide was constructed by virtual harm reduction operators, people with lived and living experience of substance use, and the research team. Fifty-two telephone interviews were conducted between November 2021 and April 2022 by two evaluators (SJ and LA) from *ThreeHive* (a third-party research organization) with master’s level training in qualitative methods. Each interview ranged from 20 to 60 min in length, and all participants were provided with a brief information package about the various virtual harm reduction services prior to the interviews. *TapeACall* and a third-party transcription service was used to record and transcribe the interviews, respectively. Honorariums of $50.00 CAD were only granted to people who use substances. All information provided by participants was kept confidential and stored on a secure server. No participant was excluded during or after the completion of interviews.

### Coding and analysis

The major themes that pertained to the perceptions of key interest groups toward virtual services and SCS were identified using grounded theory and inductive thematic analysis [26, 27]. The two evaluators who conducted the interviews coded the transcripts using *Dedoose* software. The first three transcripts were examined together and then analyzed independently afterwards. Any discrepancies that arose during this process was resolved with the principal investigator (MG). Once the initial coding was complete, the two evaluators reviewed a representative sample of coded quotations for each theme with the consulting project manager (KM). Interviews were conducted until thematic saturation was reached across all key interest groups. Data triangulation and theme checking were performed by consulting people with lived experience, overdose response hotline and app service operators, researchers, and interested government officials.

The results were reported using the Consolidated Criteria for Reporting Qualitative Research (COREQ) checklist. This study complies with Tri-Council Policy Statement for Ethical Conduct for Research Involving Humans (TCPS 2) and received approval from University of Calgary Conjoint Health Research Ethics Board (REB21-1655).

## Results

Out of the total 52 individuals interviewed, 25 identified as individuals with lived experience of substance use, 5 as family members of people with lived experience, 10 as healthcare providers, 6 as community harm reduction workers, and 6 as virtual harm reduction operators. Forty-five interviewees resided in an urban area and the remaining 7 were from a rural community. Further details regarding the area of residence are shown in Table 1. The demographic information of people who use substances and the family members is available in Table 2. The interviews elucidated the following main themes regarding the perceptions of key interest groups towards hotline/app-based and in-person services.

**Table 1:** Province/territory of residence of interviewees from each key interest group

	People with lived experience (*n* = 25)	Family members (*n* = 5)	Healthcare providers (*n* = 10)	Community harm reduction workers (*n* = 6)	Virtual harm reduction operators (*n* = 6)
Alberta	8	4	8	3	2
British Columbia	0	0	1	0	0
Saskatchewan	0	0	0	1	0
Ontario	15	0	0	0	2
Québec	1	1	0	0	0
Nova Scotia	1	0	1	0	1
Newfoundland and Labrador	0	0	0	1	0
Nunavut	0	0	0	0	1
Yukon	0	0	0	1	0

**Table 2:** Demographic information of peopled with lived experience and family members

	People with lived experience	Family members
Mean age (SD)	38.5 (12.3)	47.8 (13.9)
Gender	56% Man (*n* = 14) 40% Woman (*n* = 10) 4% Non-binary (*n* = 1)	80% Woman (*n* = 4) 20% Man (*n* = 1)
Indigenous	20% (*n* = 5)	0

### Theme 1

**Logistical and political barriers to accessing SCS.**

### Limited hours of operation and wait times

Participants described limited hours of operation and long wait times (potentially increasing the risk of “dopesickness” or unwanted withdrawal) as deterrents to seeking SCS during times of substance use. Furthermore, those residing in rural/remote areas expressed frustrations towards difficulty in accessing SCS which are often centralized in urban downtown areas. Given these factors, participants viewed virtual spotting as a convenient alternative for keeping individuals safe during times of necessary solitary use.

*“When I lived in Vancouver*,* sometimes you’d be waiting for 45 minutes to get into a SCS and it’s not exactly convenient to wait 45 minutes when … you’ve just gotten something and you’re trying to be safe.”* (Person with lived experience).

*“I do certainly think that there are good pieces within these virtual systems around access to other information and referrals to other services. I think they would be really great for rural and remote [areas]”* (Healthcare provider).

*“And the safe consumption sites, we don’t have any where I am. So that’s not an option.”* (Person with lived experience)

### Support for different routes of substance use

Although participants acknowledged that many SCS permit injection, nasal insufflation, and oral routes of substance use, they often did not allow the use of inhalants or smoking of the products. Some participants emphasized how virtual services can support clients regardless of their routes of consumption.

*“Inhalation*,* they can use the drugs they want. You know there’s one inhalation site in all of Canada. So for them to really smoke their drugs in their home while not using alone. It gives them peace of mind.”* (Person with lived experience).

“[This] *is one of the biggest benefits for NORS and DORS is that people can use at home while they’re inhaling their substances*,* whether its methamphetamines or opioids.”* (Virtual harm reduction operator).

### Privacy concerns

The fear of being seen at a SCS was a recurring theme among people who use substances, highlighting the prevailing issue of stigma around illicit substance use. Despite efforts to make SCS more welcoming, some participants noted that they can be *“pretty intimidating to be around”* (Healthcare provider) especially if the client is not familiar with the setting. Based on this finding, virtual harm reduction services may be more appropriate for clients who may prioritize anonymity and privacy.

“*In the privacy of your own house*,* you don’t have that voice in your head saying that they could see me.”* (Virtual harm reduction operator).

*“I guess the only thing that really surprised me was, for those who are meeting someone new for the first time on a call and talking about substance use, maybe giving information about your identity, etc., it may not necessarily be trustworthy to everybody, so. Whereas going to an SCS, especially in the beginning whenever, there wasn’t as much information required, you could make up a name, yeah, lots of different things, you don’t have to get an address, certain aspects were very anonymized.”* (Person with lived experience)

### Criminalization and altercation with law enforcement

People who use substances also cited fears surrounding the risk of arrest if they encountered law enforcement on the way to a SCS. In addition, participants highlighted how overdose response hotlines and apps could be particularly beneficial in places where the political atmosphere is not supportive of such facilities. Hence, these services were perceived as a reasonable alternative to support individuals regardless of where they are located geographically, especially in heavily policed jurisdictions and provinces where possession of illicit drugs is still criminalized.

*“The drug was getting dropped off by my place*,* if the drug dealer’s coming out*,* I wouldn’t go across the city to use it. It wasn’t like a social club for me. And also*,* I’d be taking a risk*,* because drugs are criminalized*,* to get all the way down there on the train or whatever*,* sneak on the train. Just use the SCS and then risk going to jail on the way. The cops would just search you. They would just randomly search you. Especially if you had a record.”* (Person with lived experience).

“*I don’t know the exact status of our consumption sites now*,* but I know that there has been some concerns or pushback or challenges to the consumption sites*,* so I don’t know how long they’re actually going to be around for*” (Healthcare Provider).

*“Providing options for people through virtual harm reduction services can show people that you don’t have to be somewhere in order for us to care about you and your safety.”* (Person with lived experience).

#### Theme 2

**Therapeutic relationships and social connection.**

SCS was seen to nurture a greater sense of social connection and meaningful relationships with other peers, harm reduction workers, and healthcare providers compared to virtual harm reduction services as they provide an opportunity for “*organic conversations*” (Person with lived experience).

*“Camaraderie and just connection with somebody in-person which […] a lot of drug users don’t have that.”* (Person with lived experience).

*“Love having people around that I’m talking to […] it’s nice being around people who are desensitized to seeing substances in front of them, but not unaware of the risks associated, while also just maintaining a relationship with you […] really it feels like that of a friend.”* (Person with lived experience)

“*Being with somebody in person, and like interacting is way more meaningful and different… virtual apps [can] take away that human aspect.”* (Healthcare provider)

*“People can use it and make connections with the people within the site; peers as well as nurses*,* counsellors*,* support people within a Supervised Consumption site. And I think that helps with using with less stigma*,* with no stigma hopefully*,* and with no judgement. And I think those connections are one of the most important reasons or one of the most important sources of helping someone heal with whatever pain they’re dealing with and why they’re using in the first place.”* (Person with lived experience).

Relationships with the staff at SCS were seen as assisting clients meet their healthcare needs in a supportive, trauma-informed environment, and to “*heal with whatever pain they’re dealing with and why they’re using* [substances] *in the first place*” (Person with lived experience). The constant engagement between clients and service providers was thought to be beneficial to the overall, sustained well-being and health of people who use substances by regaining their trust in the public health care system. One virtual harm reduction operator, however, noted how some clients much preferred the hotline than the SCS.

“[Clients] *said they feel more closely bonded to the people – the operators on the line. In the physical supervised consumption sites*,* they’re often just being observed and there’s less interaction with staff there. So*,* they’re finding that there is more interaction with the operators on the NORS lines.*”(Virtual harm reduction operator).

#### Theme 3

**Access to additional harm reduction and social services.**

One unique benefit of SCS noted by the respondents was that they provided access to clinical services that were not always available through virtual services. For instance, SCS can be used to pick up medication and receive basic health assessments and first aid (e.g., wound care) by healthcare professionals in-person.

*“I think just that social aspect is the biggest thing. And because a lot of people are accessing healthcare services at consumptions sites so maybe they’re getting wound care or they’re picking up their other medication, like a lot of folks that I know that attend supervised consumptions sites may get their you know daily HIV medications there.”* (Healthcare provider)

Participants further indicated that SCS can directly provide clients with harm reduction supplies (e.g., sterile needles, pipes, filters and other paraphernalia). Additionally, while SCS could educate clients on safer substance use, participants indicated that virtual services could at best explain how to do so over the phone. Some participants also thought that SCS may also be able to provide clients with a safer supply of opioids to help mitigate the contaminated drug supply and offer additional services, including social services and a warm place for clients to shelter - both of which it was felt virtual services could not match.

“[SCS can provide] *meal access or access to a social worker to help them with their social security* […] *help them get ID*,* help them get housing stuff”* (Healthcare provider).

“*Well I know that it’s kind of secondary to the consumption sites but I know that places would do like safer supply where people can trade in dirty street junk for something that’s at least like from a known source that at least is supposed to be what it is. I think that side of things is huge.”* (Community harm reduction worker).

#### Theme 4

**Perceived overdose response times between SCS and virtual harm reduction services**.

Many participants regarded SCS as more adept and reliable at responding to overdoses, since they are equipped with harm reduction workers and healthcare providers who can provide prompt medical assistance. However, it was deemed that virtual services are preferable to no overdose monitoring at all.

*“I would say a disadvantage is*,* response time is much slower. compared to … brick-and-mortar SCS*,* but obviously*,* you’re comparing it to none*,* then any response time obviously is better with a virtual one than none at all”* (Person with lived experience).

*“Our community geographically, our province, it’s a nightmare, it’s spread out. I mean, in some communities, like I said, it’s all volunteer people and it’s going to take a minute to mobilize them if someone’s in the house overdosing whereas if someone was, say, in the capital city or in any city in an overdose prevention site with all of that stuff’s just an arm’s reach away.”* (Community harm reduction worker)

*“The one barrier I would have about using virtual services, is them actually getting help in time. And that’s only – and I didn’t think about this one – but I know in Alberta we’ve changed our – the Emergency – the 911 calls now, they don’t come – before it used to be in your own local area and now it’s been centralized. We’ve heard lots of complaints about the – when they’re placing the Emergency call from the Call Centre, people aren’t able to find out where actually they are supposed to go. And so that I think would be a detriment, if Emergency Services aren’t able to get there in time.”* (Person with lived experience)

## Discussion

This study represents one of the first qualitative examinations of the strengths and gaps in the provision of SCS and virtual harm reduction through the lens of various key interest groups in Canada. It expands on previous studies that have examined the phenomenon of spotting and the barriers to accessing harm reduction services for people who use substances [7, 14, 17, 28].

Our study corroborates previous works that have cited similar operational and logistical barriers to using SCS [8], which may compel people to consume substances alone [29]. For instance, limited operating hours of SCS often do not cater towards people who use substances and their drug use routines [30, 31], and this was consistent with the findings of our study. Crowding is another issue faced by physical facilities [31, 32]. The Trailer, a SCS in Ottawa has been seeing more than double the number of clients it was designed to accommodate [32]. A retrospective analysis of call logs from National Overdose Response Service found that more than half of the callers could not access SCS/OPS due to reasons such as: no physical availability of SCS/OPS at the user’s location, local facility being unable to support a specific route of substance consumption, and the time of substance use being outside of SCS/OPS operating hours [7]. The current study has detailed how hotline and app-based overdose response services may be able to fill some of these gaps by providing 24/7 support especially when SCS is not a feasible option [33, 34].

Inhalation and smoking substances are becoming more common and preferred route of consumption over injection [35, 36]. This creates a challenge as only a small proportion of SCS in Canada offer inhalation support due to occupational safety hazards [37, 38]. With the increasing prevalence of crystal methamphetamine inhalation, this gap must be addressed by carefully considering its feasibility and acceptability [39]. That being said, overdose response hotlines and app services can still support individuals regardless of their preferred routes of administration at no risk to the service personnel.

Interview responses especially from people with lived experience captured concerns regarding privacy as one of the barriers to seeking in-person harm reduction services. This is a well-warranted concern given that substance use disorder is still a heavily stigmatized health status in North America, and many report not wanting to be seen by someone they know while using [30]. There were also concerns around policing from respondents who resided in jurisdictions where illicit substance use was criminalized. It has been well documented in the literature that people who use substances are reluctant to call or seek emergency medical services due to legal consequences such as arrest or losing child custody despite Good Samaritan Laws [40, 41]. Although virtual services may still contend with issues surrounding privacy [42], findings from our study suggest that virtual services are perceived to be more reassuring than physical sites.

Finding a sense of community and building therapeutic relationships was considered a unique and valuable aspect of SCS. SCS have been previously shown to be a means to break down these barriers and reshape the perceptions of peers towards health and social services [43]. While automated services like DORS and LifeGuard may be more limited in this regard, it has increasingly become a norm for peer-operated hotline services (e.g., NORS) to also facilitate social connection by providing mental health and peer support [44–48]. Despite this, there is still a utility for timer-based automated services for those who wish to have added privacy or have difficulty speaking about their substance use [44].

Respondents also highlighted how SCS provided necessary services that were lacking or unfeasible through virtual modalities. For instance, some sites are embedded in already existing health centers that not only provide immediate harm reduction support but also primary medical care and on-site opioid agonist treatment [43]. In addition, services such as wound care, clinical support, and social services are offered in a more robust fashion at physical sites, though some services are known to provide few referral services [49]. Despite this, innovative models for sterile supply distribution could be considered by overdose response hotlines and app services. Past work has suggested that mail-out kits of naloxone, needles, filters, as well as disposal containers are ways in which harm reduction services can be provisioned without a need for physical location [50].

While virtual services require activation of emergency services in the event of a suspected overdose, staff at a SCS can respond immediately due to always being physically present. Concerns regarding response times of virtual services (especially in rural communities) have been expressed by healthcare professionals and virtual harm reduction workers in previous studies [17, 49]. The time required to respond to overdose can very much impact the overall outcome of opioid-induced respiratory depression, with a proportional increased risk of hypoxic brain injury or even death with delays in overdose response [51]. Indeed, there is growing evidence that demonstrate the effectiveness of these virtual technologies in preventing and averting drug-induced overdose deaths [16, 20]. The authors believe that using overdose response hotlines and app would be safer than using alone and research is ongoing to assess its safety. Virtual harm reduction providers and operators should ensure that clients are adequately informed about the limitations of these services in terms of overdose response.

Given the increasingly toxic supply of opioids driving the overdose crisis [52], virtual services may be a much-needed service to equitably serve a large proportion of peers who do not have access to immediate spotting or other overdose prevention services [19, 42, 53]. As mentioned previously, the scalability of virtual services compared to SCS especially amidst political or community opposition makes a strong case for these service as a reasonable adjunctive option. For example, the availability of safe injection facilities and other harm reduction services in countries like United States varies greatly by jurisdiction [54, 55], and overdose response hotlines such as SafeSpot and Never Use Alone provide invaluable safeguard for people who use substances [56, 57]. While the data regarding the effectiveness and safety of these services requires continuous monitoring and examination, preliminary evidence indicates that virtual services could still be used to support solitary use of substances, prevent overdose, and foster a sense of community.

### Strengths and limitations

One strength of this study is having a relatively large sample size of individuals recruited across Canada and representing the perspectives of various key collaborators. The convenience and snowball sampling strategies used may have limited the diversity of the participants, especially those with less knowledge of virtual services. Much of our findings were also focused on hotline versions of virtual harm reduction services as opposed to automated services and wearable sensors/buttons, due to only a minority of peers having experience with them. In addition, all participants were required to have access to a mobile device or telephone and communicate effectively in English, which may have biased our sample to anglophone populations and excluded some groups, notably recent immigrants and refugees. While our study explored the potential differences between the SCS and virtual services, uptake preferences and safety were not measured quantitatively. Lastly, an examination of differences in formal virtual “spotting” methods (e.g., overdose response hotline and apps), and informal “spotting” was not examined and would be worth exploring in future studies.

## Conclusions

The findings of this preliminary study have important implications for understanding how virtual overdose response services and SCS can complement each other and understand where and in what context one service may be more applicable, acceptable, and useful than the other. These findings will further inform implementation and improvement of these services going forward.

## Data Availability

The data that support the findings of this study are available on request from the corresponding author, M.G. The data are not publicly available due to the sensitivity of substance use and interview transcripts containing information that could compromise the privacy of research participants.
